# *In Situ* Bioorthogonal Conjugation of Delivered Bacteria with Gut Inhabitants for Enhancing Probiotics Colonization

**DOI:** 10.1021/acscentsci.2c00533

**Published:** 2022-08-25

**Authors:** Wen-Fang Song, Wei-Qin Yao, Qi-Wen Chen, Diwei Zheng, Zi-Yi Han, Xian-Zheng Zhang

**Affiliations:** Key Laboratory of Biomedical Polymers of Ministry of Education & Department of Chemistry, Wuhan University, Wuhan 430072, P. R. China

## Abstract

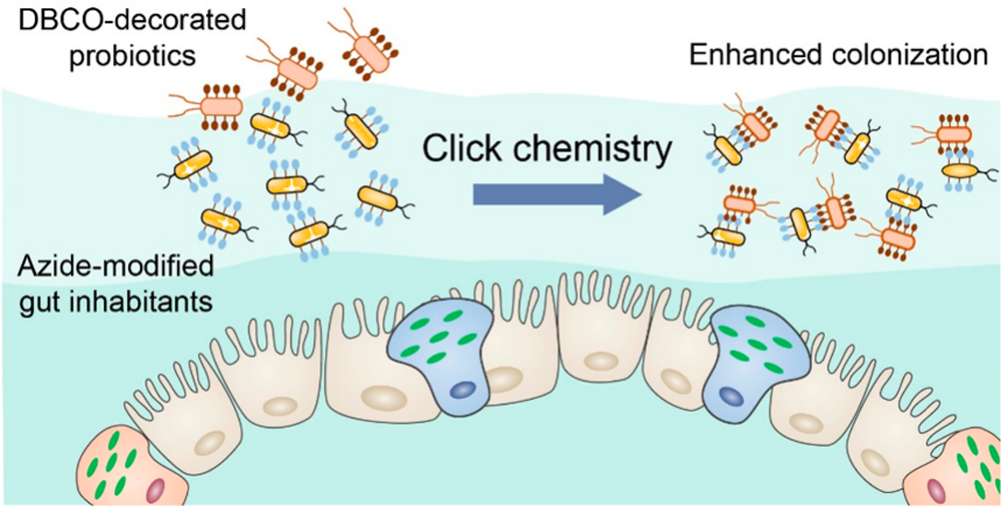

Clinical treatment efficacy of oral bacterial therapy has been largely limited by insufficient gut retention of probiotics. Here, we developed a bioorthogonal-mediated bacterial delivery strategy for enhancing probiotics colonization by modulating bacterial adhesion between probiotics and gut inhabitants. Metabolic amino acid engineering was applied to metabolically incorporate azido-decorated d-alanine into peptidoglycans of gut inhabitants, which could enable *in situ* bioorthogonal conjugation with dibenzocyclooctyne (DBCO)-modified probiotics. Both *in vitro* and *in vivo* studies demonstrated that the occurrence of the bioorthogonal reaction between azido- and DBCO-modified bacteria could result in obvious bacterial adhesion even in a complex physiological environment. DBCO-modified *Clostridium butyricum* (*C. butyricum*) also showed more efficient reservation in the gut and led to obvious disease relief in dextran sodium sulfate-induced colitis mice. This strategy highlights metabolically modified gut inhabitants as artificial reaction sites to bind with DBCO-decorated probiotics via bioorthogonal reactions, which shows great potential for enhancing bacterial colonization.

## Abstract 2

Gut inhabitants with metabolic decoration of azido groups are reported as target sites for dibenzocyclooctyne-modified probiotics to recognize and adhere through bioorthogonal reactions for improved probiotics colonization in the gut and enhanced inflammatory bowel disease therapy.

## Introduction

The gut microbiota inhabiting in the gastrointestinal (GI) tract of human contain more than 10^13^ bacterial communities.^1,2^ Accumulating evidence indicates that the gut microbiota play an essential role in regulating human health and diseases.^3−5^ Probiotics have been widely administrated orally to influence and modulate microbiome compositions, which is regarded as a promising way to prevent and treat disease.^6−9^ Unfortunately, biological challenges such as low availability and insufficient retention in the GI tract during oral delivery have largely limited the clinical translation of probiotics-delivery technologies.^10−12^ A number of strategies such as chemical surface modification, genetical engineering, and synthetic material encapsulation have been explored to enhance probiotics colonization and disease treatment efficiency.^13,14^ However, these approaches inevitably suffer from low viability, complex preparation steps, and indirect contact with intestinal mucosa, which results in inefficient survival and limited colonization of probiotics in the gut.^15,16^

With in-depth understanding of bacterial metabolic engineering, amino acid-based metabolic decoration has recently been regarded as one of the most powerful methods for gut microbes related research.^17,18^d-Amino acids (DAAs) as building blocks of bacterial peptidoglycans (PGNs) are utilized by bacteria for PGN biosynthesis. Unnatural d-amino acids also could be well-tolerated by transpeptidase involved in PGN construction and specifically incorporated into bacterial surfaces via cellular biosynthetic machinery, which suggests the possibility to decorate bacteria with functional molecules.^19,20^ On the basis of the metabolic characteristics of bacteria, special groups such as azido groups could be externally introduced into gut inhabitants precisely as an artificial target site, followed with bioorthogonal reactions.^21,22^ Considering that bioorthogonal reactions are able to rapidly occur in complex biological environments, metabolic decoration combined with bioorthogonal reactions have realized selectively targeting and tracking of bacteria *in vivo*.^23,24^ For example, bacteria with metabolic unnatural amino acid labeling could be specifically targeted and killed by clickable Janus magnetic nanoparticles through bioorthogonal reactions.^25^ Given the high selectivity and stability of chemical reactions, metabolic decoration of gut microbes with chemical groups as target sites for subsequent recognition is expected to develop an effective strategy to improve the colonization of probiotics.^26,27^

**Figure 1:**
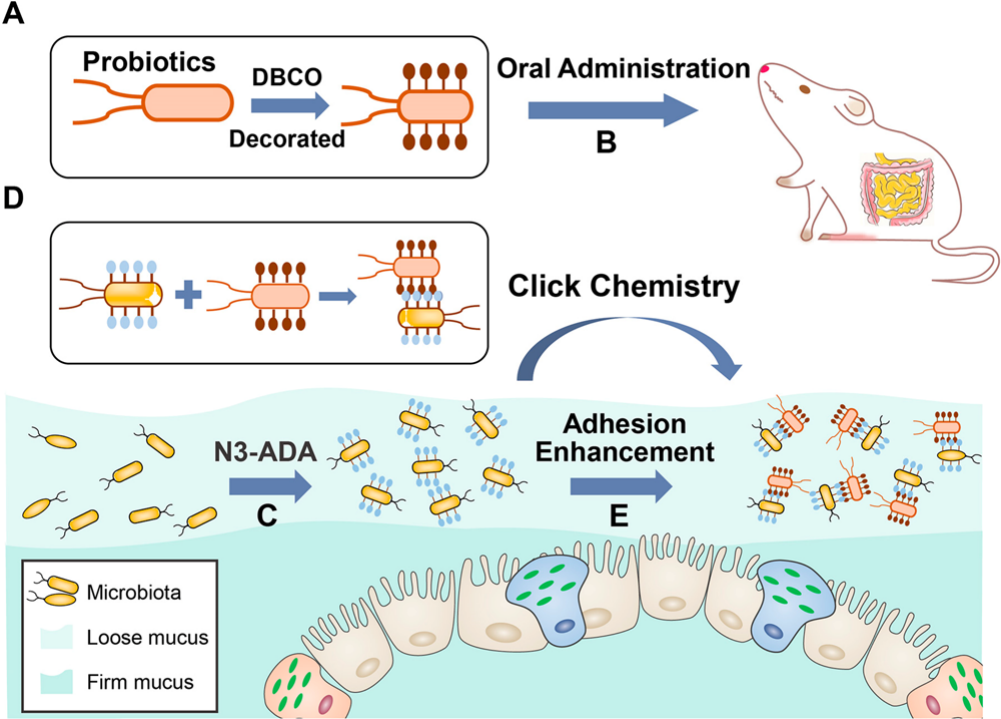
Schematic illustration of bioorthogonal-mediated bacterial delivery to enhance probiotics colonization in the gut. (A) DBCO group-modification of probiotics *in vitro*. (B) Oral administration of probiotics to mice. (C) Azido group-decoration of gut inhabitants by N3-DAA. (D) Bioorthogonal conjugation of delivered probiotics with gut inhabitants via click chemistry. (E) Enhanced adhesion of probiotics in the gut with the occurrence of the bioorthogonal reaction.

Here, we report a bioorthogonal-mediated delivery strategy that relies on metabolic amino acid engineering and bioorthogonal click chemistry (Figure 1). First, azido-modified d-alanine (N3-DAA) was metabolically incorporated into PGN of gut inhabitants for azido decoration.^28,29^ Probiotics waiting to be delivered were modified with dibenzocyclooctyne (DBCO) through amide bonds. Subsequently, DBCO-decorated probiotics were orally delivered into the GI tract of mice, followed by bioorthogonal reactions between probiotics and gut inhabitants for longer reservation.^30^*In vitro* experiments indicated that adhesion and aggregation between azido- and DBCO-modified bacteria were significantly enhanced via bioorthogonal mediation. It was also demonstrated that *in vivo* a rapidly induced bioorthogonal reaction led to conjugation of functionalized probiotics and gut inhabitants, which significantly improved the colonization efficiency of probiotics. In a dextran sodium sulfate (DSS)-induced colitis mice model, bioorthogonal-mediated delivery of *Clostridium butyricum* (*C. butyricum*) achieved efficient colonization of *C. butyricum* in the mice GI tract and an effective therapeutic effect. This bioorthogonal-mediated bacteria delivery strategy utilized metabolically labeled gut inhabitants as artificial reaction sites for *in situ* bioorthogonal conjugation with delivered probiotics, which provides a promising strategy for enhanced delivery and colonization of probiotics.

## Results and Discussion

### Azido- and DBCO-Decoration of Bacteria

**Figure 2:**
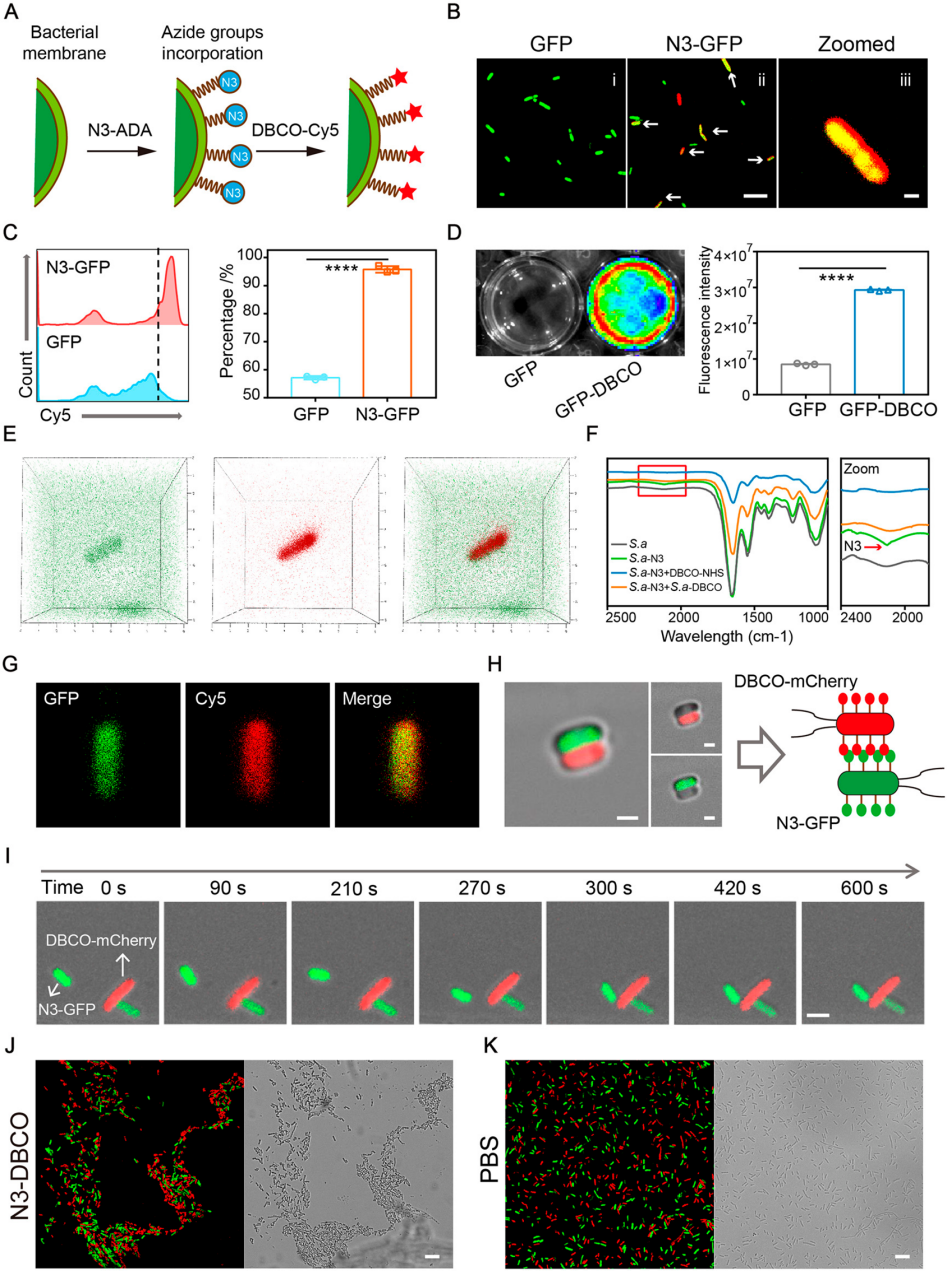
Azido and DBCO groups decorated on the bacterial wall enabled bacterial adhesion. (A) Azido decoration of GFP-labeled *E. coli*. (B) Representative confocal fluorescence images of GFP-labeled *E. coli* after incubation with PBS and N3-DAA (100 μM) for 6 h and labeling with DBCO-Cy5 (50 μM, red) for 2 h. Scale bar in (ii) 10 μm; Scale bar in (iii) 1 μm (*n* = 4). (C) Flow cytometry for quantification of the azido-decoration capacity of *E. coli* (GFP-labeled) after coincubating DBCO-Cy5 with GFP and N3-GFP for 30 min (*n* = 3). (D) Representative fluorescence images of GFP-DBCO (GFP- and DBCO group-labeled *E. coli*) and GFP after coincubating with N3-Cy5. The quantitative fluorescence intensity is also shown on the right. (E) 3D fluorescence images of DBCO-GFP by CLSM. (F) FTIR characterization of azido groups modified on bacteria before and after the occurrence of click chemistry between N3-*S. a.* and DBCO-*S. a.* (G) Fluorescence images of DBCO-GFP after incubating with N3-Cy5. (H) CLSM images of bacterial adhesion between N3-GFP (azido- and GFP-decorated MG1655) and DBCO-mCherry (DBCO- and mCherry-decorated MG1655). Scale bar, 1 μm. (I) Real-time monitoring of the adhesion of N3-GFP (azido- and GFP-labeled *E. coli* MG1655) and DBCO-mCherry (DBCO- and mCherry-labeled *E. coli* MG1655) by confocal fluorescence microscopy. Scale bar, 1 μm. (J) Bioorthogonal-mediated bacterial aggregation among N3-GFP and DBCO-mCherry. Scale bar is 10 μm (*n* = 8). (K) Bacterial aggregation among GFP and mCherry. Scale bar is 10 μm (*n* = 8). Significant differences were assessed in (D) using one-way ANOVA (Turkey; ****P ≤ 0.0001; n.s., not significant) and using the *t* test in (B). The mean values and SEM are presented.

DAAs are distributed in most bacteria as essential building blocks of bacterial PGN. Analogues of DAAs are well-tolerated by the enzymes related to PGN construction.^31^ To incorporate azido functional groups into the newly synthesized PGN, azido-modified d-alanine (N3-DAA) was added to the bacterial culture medium. Bacteria decorated with azido functional groups were subsequently incubated with DBCO-Cy5 for another 2 h (Figure 2A). To visualize interactions between functionalized bacteria, we expressed green fluorescence protein (GFP) and red fluorescence protein (mCherry) into *E. coli* MG1655, respectively. To confirm that N3-DAA was successfully incorporated into the PGN of bacteria, a fluorescence colonization assay was carried out by super-resolution confocal microscopy. As shown in Figure 2B, red fluorescence of DBCO-Cy5 was well localized on the outer surface of azido-decorated *E. coli* (GFP-labeled). As a comparison, untreated GFP-labeled *E. coli* in the GFP group showed almost no red fluorescence combing on the bacterial wall. Flow cytometry analysis was tested to similarly examine whether N3-DAA was incorporated into the PGN of bacteria. Flow cytometry quantification of red fluorescence is also shown in Figure 2C.

DBCO groups were functionalized on the surface of bacteria by coculturing of bacteria with DBCO-PEG2000-NHS via the conjugate chemistry method. DBCO-modified GFP (GFP-labeled *E. coli*) was subsequently incubated with N3-Cy5 for 2 h. The fluorescence images of GFP and GFP-DBCO were captured by the IVIS system, and the quantitative fluorescence intensity is also shown in Figure 2D. Bacteria in the GFP-DBCO had significant red fluorescence due to the click reaction with N3-Cy5, indicating that DBCO groups were well labeled on bacteria. A CLSM imaging system was also utilized for confirming whether DBCO groups were conjugated to the surface of bacteria. As shown in Figure 2E,G, both 3D fluorescence colonization images and 2D fluorescence images indicated that red fluorescence of Cy5 was displayed on the surface of GFP-labeled *E. coli*. In Figure S1, the OD600 changes of azido- and DBCO-modified bacteria through continuous monitoring are given. Bacterial intensities in N3-GFP and DBCO-GFP showed no significant difference with that in the PBS group, which indicated that azido and DBCO functionalization had little effect on the biological properties of bacteria.

Collectively, these results illustrated that bacteria cocultured with N3-DAA would be modified with azido groups and adhere with DBCO-Cy5 accurately via bioorthogonal reactions. Rapid bioorthogonal reactions were induced between azido and DBCO functional groups with forming stable chemical bonds. We therefore predicted that the bioorthogonal reaction induced between azido-decorated bacteria and DBCO functionalized bacteria would be able to mediate cell–cell adhesion.

### Bioorthogonal-Mediated Binding Enables Bacteria Cell–Cell Adhesion

Considering that azido and DBCO groups could induce rapid chemical reactions, we estimated that azido-modified bacteria could adhere to DBCO-functionalized bacteria through bioorthogonal-mediated interactions. As shown in Figure 2I, a real-time fluorescence colocalization assay was conducted by super-resolution microscopy to visualize interactions between azido- and DBCO-modified bacteria. After over 10 min of continuous observation, azido-modified bacteria (GFP-labeled) came close and adhered to DBCO-modified bacteria (mCherry-labeled). Meanwhile, azido-modified bacteria (GFP-labeled) and DBCO-functionalized bacteria (mCherry-labeled) were mixed at a ratio of 1:1 for 2 h incubation to investigate bioorthogonal-mediated bacterial aggregation. Compared with the control group, obvious gathering of green fluorescence (GFP-labeled) and red fluorescence (mCherry-labeled) was observed in the N3-DBCO group (Figure 2J and Figure 2K). All of the results illustrated that bacterial adhesion was enhanced between azido-modified bacteria and DBCO-functionalized bacteria though bioorthogonal mediations (Figure 2H). To explore whether the click reaction was induced between azido-modified bacteria and DBCO-modified bacteria during bacterial aggregation, Fourier transform infrared spectroscopy (FTIR) characterization of azido groups was performed. An azido characteristic peak at 2102 cm^–1^ is shown in the azido-modified bacteria, which was not observed in other groups (Figure 2F). The disappearance of the azido characteristic peak in *S.a*-N3+*S.a*-DBCO showed the occurrence of click reactions between azido-modified and DBCO-modified bacteria. To explore the changes in the zeta potential before and after bacterial adhesion, we analyzed the potential of blended bacteria. The data of the electric potential indicated that bacterial adhesion had no significant effect on the potential of bacteria (Figure S2).

Next, to investigate the applicability of the method of bioorthogonal-mediated bacterial adhesion, we detected the adhesion effects of different bacteria strains after functional modification of azido and DBCO groups. Bacterial adhesion between azido-decorated *E. coli* (Cy5-labeled) and DBCO-modified *Peptostreptococcus anaerobius* (*P. anaerobius*; FITC-labeled) was observed under a super-resolution microscope. Compared with the untreated group, bacterial adhesion among bacteria modified by the click chemistry functional group was significantly enhanced (Figure S3). The same result was also observed in the pictures taken by a transmission electron microscope (TEM) (Figure S4). At the same time, we tested the bacterial adhesion between azido-modified *Staphylococcus aureus* (*S. aureus*) and DBCO-functionalized *E. coli* MG1655. As expected, fluorescence colonization analysis revealed that bioorthogonal-mediated bacterial adhesion was significantly enhanced (Figure S5).

### *In Vitro* Visualizing Bioorthogonal-Mediated Bacterial Coaggregation

**Figure 3:**
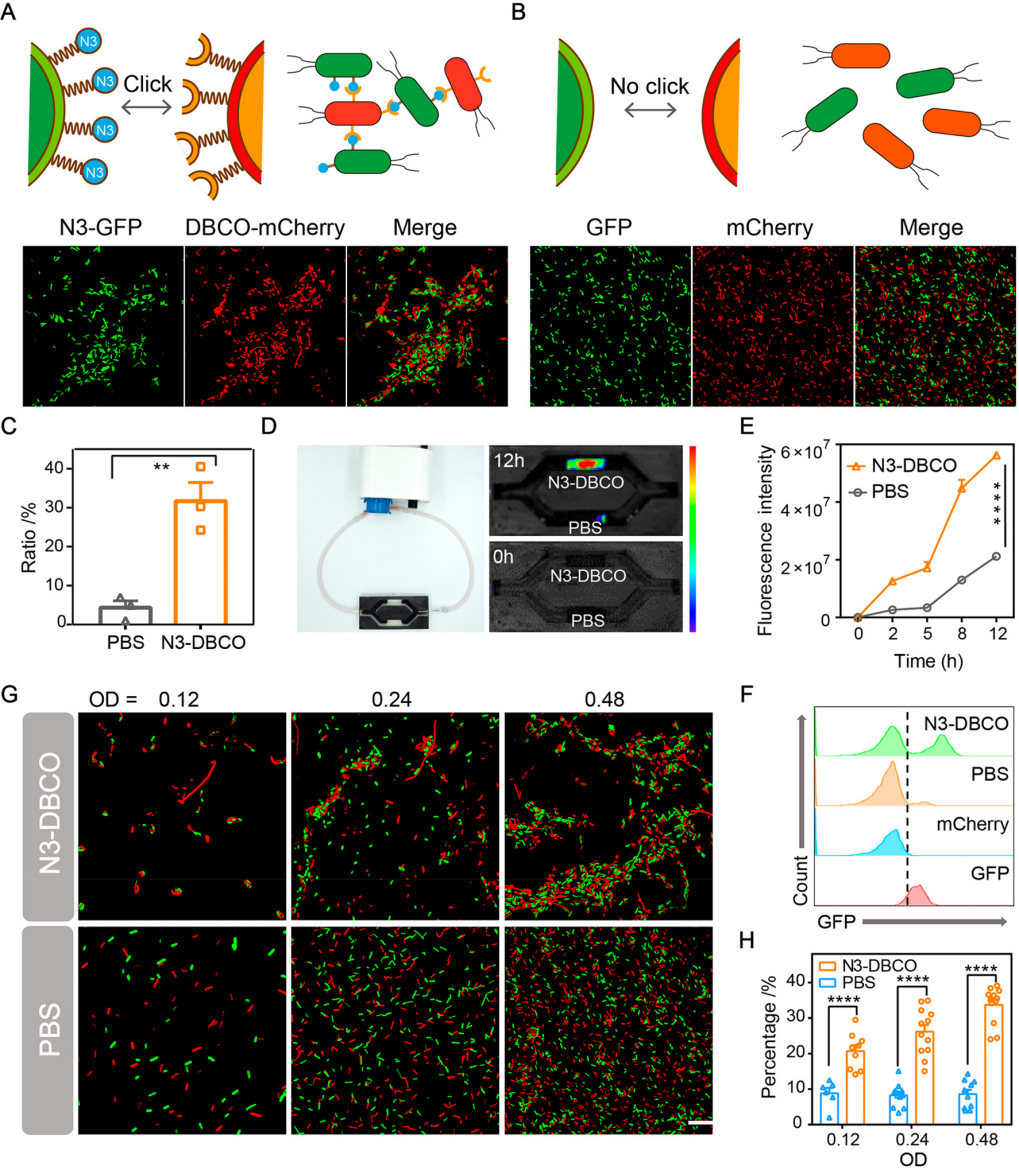
*In vitro* characterization of bacterial aggregation induced by bioorthogonal reactions. (A) and (B) Representative confocal fluorescence images of bioorthogonal-mediated bacterial adhesion between N3-GFP + DBCO-mCherry and GFP + mCherry at a 1:1 ratio (OD = 0.48). Scale bar, 1 μm (*n* = 3). (C) Aggregation ratio analysis by the particle analyzing function of ImageJ. (D) *In vitro* bioorthogonal-mediated binding effect of N3-GFP and DBCO-mCherry in a flow environment. DBCO-mCherry was added to the circulating fluid, and the red fluorescence (mCherry) of hydrogel containing N3-GFP was monitored by IVIS (*n* = 3). (E) Fluorescence intensities at different time points. (F) Flow cytometry analysis for evaluating bacterial aggregation caused by bioorthogonal reactions (*n* = 3). (G) Representative confocal fluorescence images and (H) aggregation ratios of 1:1 mixed cocultures at different bacterial densities (*n* = 6). Scale bar, 1 μm. Significant differences were assessed in (C), (E), and (H) using the *t* test (**P ≤ 0.01; ****P ≤ 0.0001). The mean values and SEM are presented.

To visualize bioorthogonal-mediated bacterial adhesion, the aggregation degree of bacteria modified with click chemistry functional groups at different densities was observed by super-resolution confocal microscopy. As shown in Figure 3A,B, the green fluorescent field (azido-modified bacteria), the red fluorescent field (DBCO-functionalized bacteria), and the fluorescent overlay field were exhibited. The aggregation ratio of click chemistry-functionalized bacteria was quantitatively analyzed by ImageJ. We evaluated the aggregation ratio of bacteria in the N3-DBCO group and control group. First of all, we calculated the number of bacteria in the red fluorescence and green fluorescence images respectively according to the size of bacteria. Then, the aggregation degree of the fluorescence superposition field was analyzed (three times the area of a single bacteria was defined as aggregation).^32^ Quantitative data indicated that aggregation rate of bacteria modified by the click chemistry functional group was 31%, while the aggregation rate of bacteria in the control group was about 5% (Figure 3C). These data illustrated the enhanced effect of bioorthogonal click chemistry on bacterial aggregation. Meanwhile, we explored the effect of different bacterial densities on the bacterial adhesion mediated by bioorthogonal click chemistry. We mixed different functionalized bacteria at ratios of 1:1, 2:2, and 4:4 and observed their aggregation under a super-resolution confocal microscope. As shown in Figure 3G and Figure S6, bacteria mixed at various densities had obvious bacterial aggregation compared with the control group. Quantitative analysis of the aggregation degree between functionalized bacteria revealed that the adhesion rate of bacteria could be significantly improved with the increase in bacterial density, while bacterial adhesion in the control group was not affected by the mixing ratio of bacteria (Figure 3H).

To further simulate bioorthogonal-mediated bacterial aggregation in a complex environment, we used a microfluidic device to simulate the flow environment of the intestine. The upper and lower chambers of the microfluidic device were placed with hydrogels loaded with azido-decorated bacteria and untreated bacteria, respectively. The external circulating fluid containing DBCO-functionalized bacteria (mCherry-labeled) flowed in the circulating pump at a certain speed. Fluorescence of the microfluidic device was monitored by fluorescence imaging for 12 h. As displayed in Figure 3D, the upper chamber of the microfluidic device showed obvious red fluorescence over time, while the lower chamber was not observed. Quantitative analysis of fluorescence intensity indicated that red fluorescence intensity of the upper chamber increased significantly over time, while the fluorescence intensity of the control group may be slightly increased due to the retention of bacteria by the hydrogel (Figure 3E). These results indicated that azido-modified bacteria could adhere to DBCO-functionalized bacteria through click chemistry in the flow environment, while the untreated bacteria had no obvious bacterial adhesion.

At the same time, flow cytometry was conducted to quantitatively analyze and evaluate the bacterial adhesion. Compared with other groups, azido-decorated bacteria (GFP-labeled) showed obvious red fluorescence binding, while almost no red fluorescence was found on bacteria in the untreated group (Figure 3F). The above results showed that azido-modified bacteria and DBCO-functionalized bacteria had specific adhesion, while no obvious bacterial adhesion was found between bacteria of the control group.

Overall, all of the above results indicated that significant aggregation could occur between azido-modified bacteria and DBCO-functionalized bacteria via bioorthogonal reactions. Changing the densities of bacteria had almost no significant effect on the aggregation of bacteria in the PBS group, while bacterial aggregation rate showed little improvement as the bacterial density increased. The *in vitro* intestinal simulation experiment illustrated that click chemistry-mediated adhesion could still be carried out in a complex flowing environment. Modulating bacterial binding via click-chemical functional group modification is expected to be a potential strategy for enhancing bacterial delivery in the intestinal tract.

### *In Vivo* Tracking Colonization of DBCO-Modified Bacteria

**Figure 4:**
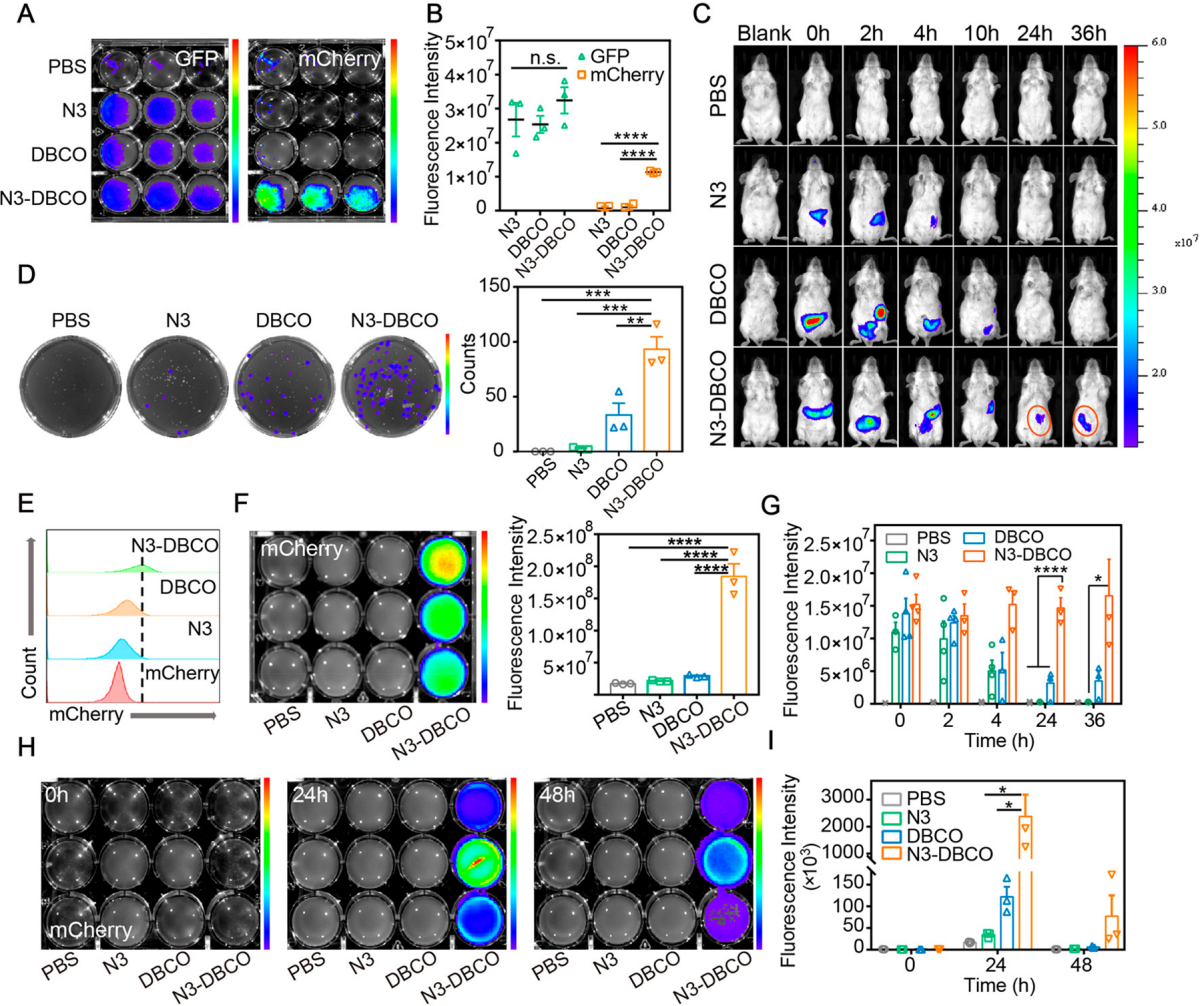
*In vivo* enhanced bacterial retention with bioorthogonal mediation. (A) Fluorescence imaging of GFP and mCherry in mouse feces after 20 h culture in LB medium. Mice in different groups were gavaged with N3-GFP, GFP + DBCO-mCherry, N3-GFP + DBCO-mCherry. Feces of mouse in different groups were collected 6 h after gavage (*n* = 3). (B) Quantitative analysis of GFP and mCherry fluorescence intensity in mice feces of different groups. (C) Representative IVIS images for evaluating bacterial retention in the GI tract of mice mediated by biorthogonal reactions (*n* = 3). (D) Bacterial colony-forming units of mCherry in LB agar plates for quantifying the number of mCherry in mice colon tissues. Fluorescence imaging of mCherry is shown on the plates. Quantitative analysis of fluorescence intensity is also shown on the right (*n* = 3). (E) Flow cytometry analysis for quantification of mCherry in mice colon tissues. (*n* = 3). (F) *In vitro* fluorescence imaging of mCherry in mice colon tissues after 20 h culture in LB medium. Quantitative analysis of mCherry fluorescence intensity is shown on the right (*n* = 3). (G) Quantitative analysis of *in vivo* mCherry fluorescence intensity at different time points. (H) Representative IVIS images of cultured mixture with feces for evaluating bacterial colonization over 48 h. Feces were collected at different time points after the gavage of mCherry (*n* = 3). (I) Quantification of fluorescence intensities. Significant differences were assessed in (B), (D), (F), (G), and (I) using one-way ANOVA (*P ≤ 0.05; **P ≤ 0.01; ***P ≤ 0.001; ****P ≤ 0.0001; n.s., not significant). The mean values and SEM are presented.

We explored the application of click chemistry-mediated bacterial adhesion to bacterial delivery in the mouse intestine. The gut microbiota of mice were cleared after 3 days of antibiotic cocktail gavage.^33^ Subsequently, azido-modified bacteria (GFP-labeled) were colonized in the intestine with 3 days gavaged. Then, DBCO-functionalized bacteria (mCherry-labeled) were delivered to the intestine of mice. To investigate the colonization of bacteria modified with click chemistry functional groups, we collected mice feces of different groups in 6 h after the delivery of DBCO-modified bacteria. Fluorescence imaging analysis was performed after 20 h of culture of mouse feces *in vitro*. As shown in Figure 4A, green fluorescence was observed in the culture medium of each group, while obvious red fluorescence was only observed in the N3-DBCO group. We further quantified and compared the green fluorescence and red fluorescence of each control group (Figure 4B). The analysis of *in vivo* imaging fluorescence intensities indicated that bacteria (GFP-labeled) were significantly colonized in mice, while the subsequently delivered bacteria (mCherry-labeled) were only colonized in mice of the N3-DBCO group. Meanwhile, we spread the mouse fecal grinding liquid on the LB culture plate containing ampicillin. After 24 h of incubation, the plate was subjected to red fluorescence imaging using fluorescence imaging technology, and the number of colonies was counted (Figure 4D). The above results illustrated that bioorthogonal-mediated bacterial adhesion allowed delivered bacteria to colonize in the GI tract of mice for a longer time.

To more intuitively observe the bioorthogonal-mediated bacterial delivery in the intestines of mice, we monitored the red fluorescence in the intestine of mice for 36 h. As shown in Figure 4C, red fluorescence in the intestine of mice in each group was significantly enhanced in the first 4 h after the delivery of bacteria (mCherry-labeled). Compared with the control group, red fluorescence at the intestine in the click chemistry-mediated group (N3-DBCO) lasted until 36 h, while almost no red fluorescence was detected in the control groups. Quantitative analysis of red fluorescence intensity revealed that a large number of DBCO-modified bacteria (mCherry-labeled) still colonized in the intestine of mice in the N3-DBCO group after 36 h of bacterial delivery (Figure 4G).

To explore the composition of gut microbes of mice in depth, mice were sacrificed at 36 h after bacterial delivery, and intestinal tissues were taken for grinding and culturing in LB medium *in vitro*. Through flow cytometry analysis of the culture medium, we found that massive bacteria (GFP-labeled) were colonized in the intestine of mice in each group, while abundant mCherry-labeled bacteria only were observed in mice of the N3-DBCO group at the same time (Figure 4E). The same results were confirmed in fluorescence imaging assay. Compared with the control groups, red fluorescence of culture medium supplemented with tissue homogenate in the N3-DBCO group was clearly observed, which meant delivered bacteria (mCherry-labeled) had more efficient colonization in the gut of mice in the N3-DBCO group (Figure 4F). To further investigate the long-term colonization of click chemistry-mediated bacterial delivery in the intestine of mice, we conducted continuous observations of red fluorescence in the intestine of mice. Mice in different groups were imaged by the IVIS system at continuous time points (Figure S7). As shown in Figure S8, colons of mice in each group were also collected and imaged by the IVIS system at different time points. Fluorescence of mice and colons in the N3-DBCO group were obviously detected at 24 and 48 h, while the same fluorescence was not seen in the other groups. At 0, 24, and 48 h after bacterial delivery, feces of mice were collected and cultured in LB medium. The intensities of red fluorescence in each group were detected and analyzed. As shown in Figure 4H, red fluorescence could be observed in the N3-DBCO group for 48 h, indicating that bacteria (mCherry-labeled) had been clearly colonized in the intestine of mice until 48 h. The following fluorescence quantification data also illustrated this point (Figure 4I). The above data demonstrated that bioorthogonal-mediated delivery of bacteria exhibited potential application prospects in probiotics supplementation to the intestine.

### Bioorthogonal-Mediated Bacterial Delivery Ameliorates Disease Activity in DSS-Induced Colitis Mice

**Figure 5:**
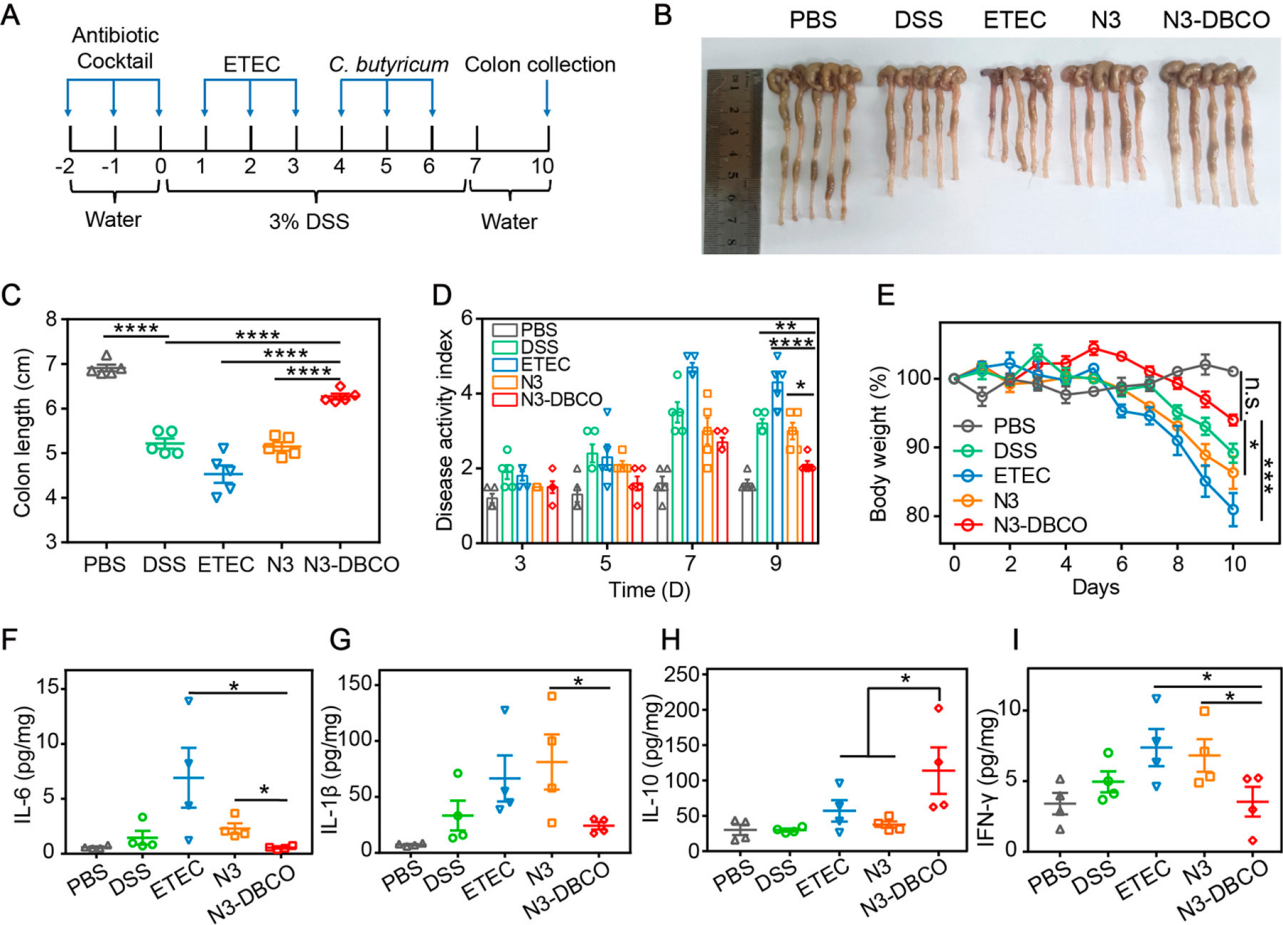
*In vivo* bioorthogonal-mediated therapy in a DSS-induced colitis mice model. (A) Schematic of administration schedule. C57BL/6 mice were provided with PBS, ETEC (10^8^ CFU per mouse), or N3-ETEC (10^8^ CFU per mouse) for 3 days after antibiotic cocktail treatment. On days 4, 5, and 6, mice were orally administered PBS, *C. butyricum* (10^8^ CFU per mouse), or DBCO-modified *C. butyricum* (10^8^ CFU per mouse). Mice were euthanized on day 10. (B) Representative photographs of mice colons with different treatments (on day 10) (*n* = 5). (C) Colon length of mice in each group (*n* = 5). (D) Disease activity index of mice feces on days 3, 5, 7, and 9 (*n* = 5). (E) Body weight changes over 10 days (*n* = 5). (F–I), IL-6, IL-1β, IL-10, and IFN-γ protein expression levels of colon tissues by ELISA (*n* = 4). Significant differences were assessed in (C), (D), and (E) using one-way ANOVA and the *t* test in (F), (G), (H), and (I) (*P ≤ 0.05; **P ≤ 0.01; ***P ≤ 0.001; ****P ≤ 0.0001; n.s., not significant). The mean values and SEM are presented.

Bioorthogonal-mediated bacterial delivery to colonize in the GI tract of mice was demonstrated previously *in vitro* and *vivo* experiments. To validate the therapeutic effects of bioorthogonal-mediated bacterial delivery in DSS-induced colitis mice, we combined clinically approved probiotics for the treatment of colitis, *C. butyricum*, and a bioorthogonal-mediated delivery strategy for the treatment of mice colitis.^34^ The overall experimental procedure is summarized in Figure 5A. Mice were given 3% (w/v) DSS in drinking water for 7 consecutive days to induce colitis. With the progression of colitis, the body weight and colon length of mice changed significantly (Figure S9). In the early stage of colitis, azido-modified enterotoxigenic *E. coli* (ETEC; ATCC 25922) were colonized and DBCO-decorated *C. butyricum* were subsequently administrated by gavage on days of 4, 5, and 6. *E. coli* ATCC 25922 is known for its ability of producing colibactin toxin to exacerbate the disease progression of colitis.^35^ In the mouse model of colitis, mice received delivery of *C. butyricum* in different ways, and the state of colitis was monitored by body weight changes of mice and pathological score of feces as shown in Figure 5E,D.^36^ Mice in the N3-DBCO group exhibited significant reduction in weight loss and improved stool pathology scores compared to the other groups. After the treatment of 10 days, mice were sacrificed, and the colons of mice in each treatment group were collected. Changes in colon length were also measured to evaluate the therapeutic efficacy (Figure 5B,C). The data of colon length indicated that bioorthogonal-mediated delivery of *C. butyricum* protected mice from DSS-induced shortening of the colon length. To investigate the efficacy of *C. butyricum* in colitis mice, the expression levels of several inflammation-associated cytokines in the colon were detected by the enzyme-linked immunosorbent assay (ELISA).^37^ As shown in Figure 5F–I, we found that the expression levels of IL-6, IL-1β, and IFN-γ in the N3-DBCO group were reduced compared with the other groups.

**Figure 6:**
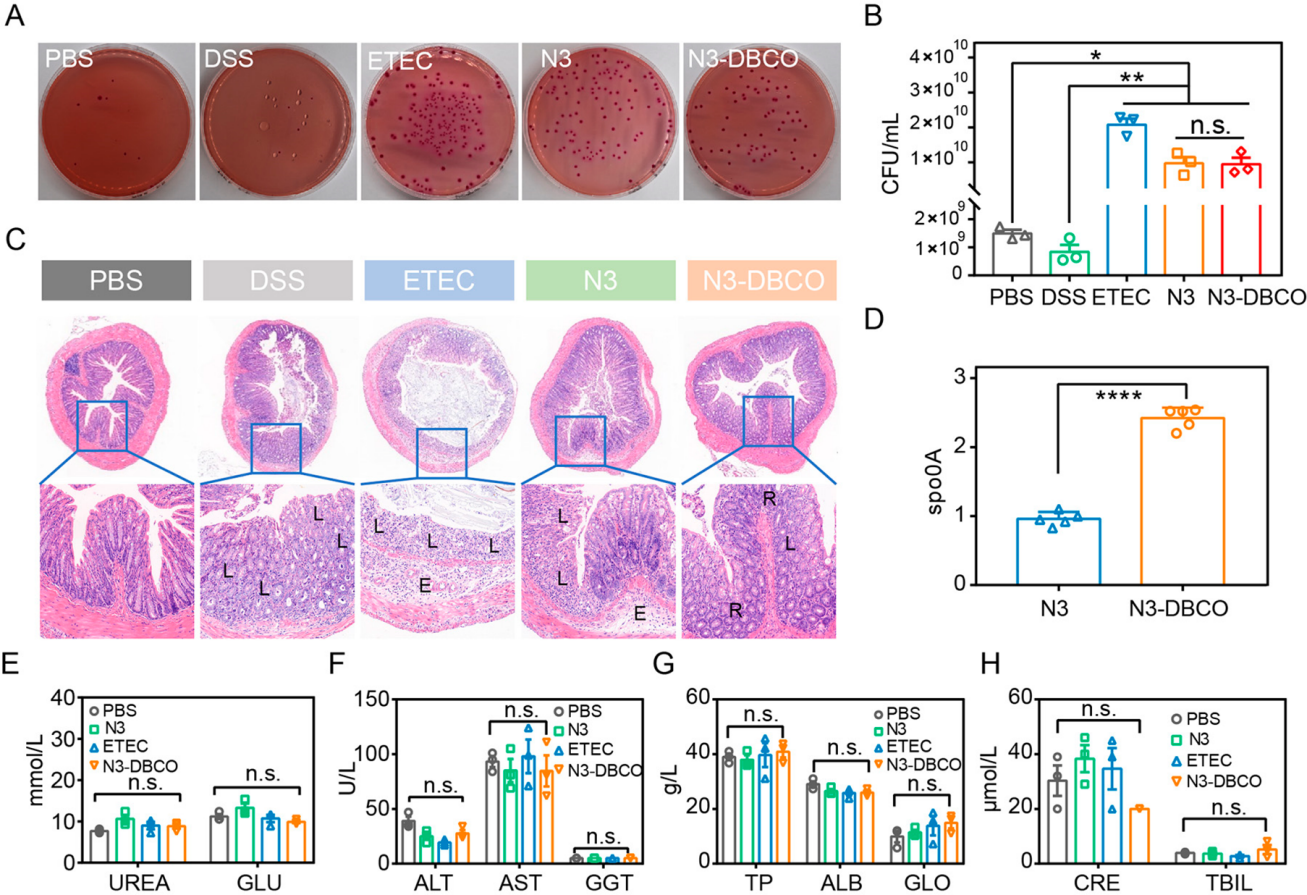
Increased abundance of *C. butyricum* and biosafety assay. (A) Bacterial colony-forming units of ETEC in chromogenic *E. coli* agar plates for indicating the colonization of ETEC in the GI tract of mice. Mice feces were collected on day 3 (*n* = 3). (B) Quantitative analysis of the number of ETEC in mice feces. (C) Representative histology of colon pathological sections of mice in different treatment groups stained with hematoxylin and eosin. Image markers indicate partial loss of the crypts (L), tissue edema (E), and recovery of crypts (R). (D) PCR analysis of colon tissues for evaluating the colonization of *C. buturicum* in the GI tract (*n* = 5). (E) Concentrations of UREA and GLU related to kidney function on the 10th day (*n* = 3). (F) Liver function related enzyme concentrations of ALT, AST, and GGT on day 10 (*n* = 3). (G) and (H) Main blood cell concentrations of TP, ALB, GLO, CRE, and TBIL on day 10 (*n* = 3). Significant differences were assessed in (D) using the *t* test and (****P ≤ 0.0001) and one-way ANOVA in (B), (E), (F), (G), and (H) (*P ≤ 0.05; **P ≤ 0.01; n.s., not significant). The mean values and SEM are presented.

At the same time, the expression levels of anti-inflammatory cytokine IL-10 in the N3-DBCO group were significantly increased. Moreover, bioorthogonal-mediated *E. coli* Nissle1917 delivery to the gut was also performed and led to obvious disease relief in DSS-induced colitis mice (Figure S10). After the treatment, the number of enterotoxigenic *E. coli* in the mouse intestines was explored using chromogenic *E. coli* agar plates. Feces of mice in each group were collected after treatment, and the number of enterotoxigenic *E. coli* in feces were counted by the plate coating method (Figure 6A,B and Figure S11). As illustrated in Figure 6D, the *Y*-axis ordinate indicated the relative expression of genes of *C. butyricum*, which represented the content of *C. butyricum* in mice colon. A significantly enhanced abundance of *C. butyricum* was observed in the mice colons of the bioorthogonal-mediated delivery group by 16S rDNA (rDNA) sequencing. At the same time, pathological sections of colon after treatments are displayed in Figure 6C. The structure of colonic epithelium and colonic tissue collapsed was disrupted severely in the mice of the DSS group and DSS + ETEC group. Compared with the other groups, improved histological appearance was observed with treatment of colonized *C. butyricum* in mice of the bioorthogonal-mediated delivery group. Finally, blood biochemistry and blood routine tests were performed to evaluate peripheral blood cells and liver and renal function (Figure 6E–H).

## Conclusion

Gut microbiota plays a critical role in preventing and treating diseases. The delivery of probiotics to regulate the composition of gut microbiota has shown great potential in the treatment of diseases. However, the complex intestinal environments have limited delivery efficiency of probiotics by influencing colonization and proliferation of bacteria in the GI tract. With the in-depth understanding of bacterial metabolic decoration, we propose a bioorthogonal-mediated bacterial delivery strategy to enhance bacterial adhesion between DBCO-functionalized bacteria and metabolic-decorated gut inhabitants for longer colonization. Metabolic decoration of gut inhabitants has exogenously introduced azido groups onto the surface of gut inhabitants as artificial target sites for subsequent recognition. Bioorthogonal click chemistry stands out for its remarkable specificity and exceptional reaction kinetics, which could enable fast and specific bacterial adhesion even in complex physiological environments.

GFP and mCherry fluorescence proteins expressed *E. coli* MG1655 were utilized to better observe and explore the interactions between azido- and DBCO-modified bacteria *in vitro*. It was also found that *in vivo* click chemistry-mediated bacterial adhesion between probiotics and gut inhabitants could improve the colonization of probiotics in the gut. Meanwhile, bioorthogonal-mediated delivery of *C. butyricum* showed significantly efficient intestinal colonization of *C. butyricum* and alleviated disease progression in a colitis mouse model. Our study highlights the potential bacterial delivery strategy that combines bacterial metabolic engineering with bioorthogonal click chemistry to counteract the complex intestinal environment and achieve enhanced delivery of bacteria in the GI tract. With the introduction of this concept, we expect that bioorthogonal-mediated bacterial delivery for regulating gut microbes and treating diseases will find great potential in clinical translation.

## Experimental Section

### Bacterial Strains

All of the above experiments used second to fourth generation bacteria. *E. coli* [American Type Culture Collection (ATCC 700926)] and *C. butyricum* (ATCC 19398) were obtained from the ATCC. *S. aureus* (ATCC 25923) was purchased from China Center for Type Culture Collection (CCTCC). Luria–Bertani (LB) broth and sporogenous medium were used for the bacterial culture. GFP and mCherry-labeled *E. coli* MG1655 were obtained by cotransforming a fluorescent protein expression plasmid (GFP or mCherry, ampicillin resistant). *E. coli*, *S. aureus*, and *E. coli* Nissle1917 were cultured in LB medium under stirring at 37 °C. *C. butyricum* was cultured in sporogenous medium at 37 °C with anaerobic incubation. GFP and mCherry-labeled *E. coli* were cultured in LB medium with 100 U/mL penicillin. All of the bacteria were purified from the colonies isolated on the plate and used for subsequent liquid culture.

### Animal Model Establishment

All animal experiments were conducted under protocols approved by the Institutional Animal Care and Use Committee (IACUC) of the Animal Experiment Center of Wuhan University (Wuhan, P. R. China). Antibiotic cocktail for three consecutive days was used to eliminate intestinal flora of mice. Delivery efficiency of *C. butyricum* with click chemistry mediation was evaluated in a colitis mice model. DSS at a concentration of 3% was used for constructing the colitis mice model with the female C57 mice of 8 weeks. Body weights of mice were recorded every day, and feces pathology scores were evaluated every 2 days.

### Materials

LB medium, SB medium, *E. coli* chromogenic medium, and agar were purchased from Guangdong Huankai Microbial SCI & Tech Co., Ltd. 1-Ethyl-3(3-(dimethylamino)propyl) carbodiimide (EDC) and *N*-hydroxysuccinimide (NHS) were purchased from Energy Chemical (Shanghai, China). DBCO-PEG2000-NHS was purchased from Pousure Biotech, Inc. (Shanghai, China). 3-Azido-d-alanine was purchased from Fluorochem., Ltd. DBCO-Cy5 was obtained from Sigma-Aldrich. Ampicillin was purchased from Macklin Inc.

### Azido Group Decoration of Bacteria

*E. coli* (GFP-labeled) and enterotoxigenic *E. coli* (ATCC 25922) were incubated in LB medium at 37 °C until the midexponential phase. N3-DAA was then added into the culture medium to a final concentration of 100 μM. After culturing for 6 h, the bacteria were washed with PBS twice and then suspended in PBS.

### DBCO Groups Decoration of Bacteria

*E. coli* (mCherry-labeled) and *C. butyricum* were incubated in LB medium and SB medium at 37 °C respectively until the midexponential phase. The bacteria were washed with PBS twice and then suspended in PBS. DBCO-PEG2000-NHS was then added into the suspension to a final concentration of 100 μM. After incubation for 2 h, the bacteria were centrifuged at a speed of 5000 rpm/min for 3 min and then resuspended in PBS.

### *In vitro* Bioorthogonal-Mediated Bacterial Selective Adhesion Assay

Azido-decorated bacteria (GFP-labeled) and DBCO-functionalized bacteria (mCherry-labeled) were resuspended in PBS and mixed in a 1:1 ratio (OD600 = 0.48). After coincubation for another 2 h, 10% paraformaldehyde was added to each bacterial suspension for fixation for 20 min to image bacteria better. Bacterial aggregation and difference in binding ability between bioorthogonal-mediated bacteria and untreated bacteria were analyzed by a confocal laser scanning microscope (CLSM). Images were acquired on CLSM for imaging the GFP (488 nm excitation) and mCherry (580 nm excitation). For each sample, 10 images were taken in the GFP and mCherry channels, respectively.

To analyze the bacterial aggregation at different densities, azido-decorated bacteria (GFP-labeled) and DBCO-functionalized bacteria (mCherry-labeled) were respectively mixed in a 1:1 ratio to a different final OD600. Bacterial aggregation was performed the same as described above. To quantify bacterial aggregation at different densities, all of the images were analyzed by ImageJ. The GFP and mCherry channels of images were merged and converted into binary images. On the basis of the Analyze Particles function in ImageJ, all of the bacteria and clustered bacteria in each image were counted. The particle size of single bacteria was acquired by the result of a particle analysis tool, and three times area of single bacteria was defined as aggregation. The aggregation ratios were calculated by the sum of clustered bacteria/sum of all bacteria.

### Real-Time Imaging of Bacterial Adhesion *in Vitro*

Azido-decorated *E. coli* (GFP-labeled) and DBCO-functionalized *E. coli* (mCherry-labeled) were suspended in PBS to a final OD600 = 0.24 and mixed in a 1:1 ratio. After coculturing for 4 h, bacteria solution was dropped on the glass slide and observed by CLSM. The real-time images were taken by CLSM over a continuous time for 10 min. As described above, the 488 nm excitation was used for imaging GFP-labeled *E. coli*, and the 580 nm excitation was used for imaging mCherry-labeled *E. coli*, respectively.

### *In Vitro* Circulating Device for Simulating the Intestinal Environment

We used a microfluidic device to simulate the physiological environment of the intestine tract. Azido-decorated *E. coli* and *E. coli* were immobilized in 1% agar hydrogel (10^8^ CFU of bacteria). Artificial intestinal juice (100 mM KH2PO4, pH 6.8) containing DBCO-decorated *E. coli* (mCherry-labeled) was cycled for 12 h at a speed of 0.85 cm/s. The IVIS system was used to measure fluorescence intensity of mCherry in the upper and lower chambers at the time points of 0 min, 30 min, 1, 2, 5, 8, and 12 h.

### Flow Cytometry Analysis of Bacterial Aggregation

Flow cytometry analyses (BD FACS Aria TM III) were performed to analyze bacterial adhesion of the bioorthogonal modification groups and untreated groups. Azido-decorated *E. coli* (GFP-labeled) was incubated with DBCO-Cy5 for 2 h and then washed with PBS twice. GFP-labeled *E. coli* was identified and then gated on the Cy5 fluorescence. Flow Jo software was used to analyze data. For the *in vitro* click chemistry-mediated bacterial adhesion assay, azido-decorated *E. coli* (GFP-labeled) and DBCO-functionalized *E. coli* (mCherry-labeled) were incubated for 2 h. GFP-labeled bacteria were identified and then gated on the mCherry fluorescence. The data were analyzed as described above. For the *in vivo* bacterial colonization assay, 100 μL of antibiotic cocktail at a concentration of 10 mg/mL was gavaged to BALB/c mice for 3 days. GFP-labeled *E. coli* (10^8^ CFU per mouse) was orally administrated for 3 days, and then mCherry-labeled *E. coli* (10^8^ CFU per mouse) was delivered. Feces of mouse in different groups were collected 24 h after bacterial delivery. Each sample was ground with 1 mL of PBS by tissue homogenizer for 3 min. Then, the sample was cultured in LB medium for 24 h, and flow cytometry analyses were as described above.

### Imaging Bacterial Colonization *in Vivo*

Antibiotic cocktail (100 μL) was gavaged to C57 mice for 3 days. Then, GFP-labeled *E. coli* was orally administrated for 3 days. Mice were divided into four groups: PBS (100 μL of PBS), N3 (azido- and GFP-labeled *E. coli*, 100 μL, 10^9^ CFU/mL), DBCO (GFP-labeled *E. coli*, 100 μL, 10^9^ CFU/mL), N3-DBCO (azido- and GFP-labeled *E. coli*, 100 μL, 10^9^ CFU/mL). On the fourth day, mCherry-labeled *E. coli* was delivered subsequently. PBS (100 μL) was orally administrated to mice in the PBS group. *E. coli* (mCherry-labeled, 100 μL, 10^9^ CFU/mL) was given to mice in the N3 group by gavage. DBCO-decorated *E. coli* (mCherry-labeled, 100 μL, 10^9^ CFU/mL) was orally administrated to mice in DBCO and N3-DBCO groups. The IVIS system was used to detect the fluorescence intensity of mCherry in mice at 0, 2, 4, 10, 24, and 36 h. Feces of mice were collected at the time points of 0, 24, and 48 h. After 2 mL of PBS was added into the sample, feces samples were homogenized by a tissue homogenizer for 3 min. 100 μL of homogenate in each sample was removed into 3 mL of LB medium for coincubation. After 4 h, the bacterial solution was added to a 12-well plate, and IVIS (PerkinElmer) was used for detecting the fluorescence intensity of mCherry.

### Bioorthogonal-Mediated Treatment Assay in a DSS-Induced Colitis Mouse Model

Mice were given antibiotic cocktail by gavage for 3 days. Then, ETEC with different decoration was orally administrated to mice for 3 days. Mice were divided into five groups: PBS (100 μL PBS), DSS (100 μL PBS), ETEC (enterotoxigenic *E. coli*, 100 μL, 10^9^ CFU/mL), N3 (azido-decorated ETEC, 100 μL, 10^9^ CFU/mL), N3-DBCO (azido-decorated ETEC, 100 μL, 10^9^ CFU/mL), respectively. Then, PBS (100 μL) was orally administrated to mice in the PBS, DSS, and ETEC groups. *C. butyricum* (100 μL, 10^9^ CFU/mL) was given to mice in the N3 group by gavage. DBCO-decorated *C. butyricum* (100 μL, 10^9^ CFU/mL) was orally administrated to mice in the N3-DBCO group. Mice in each group (except PBS) were fed with 3% DSS during day 0 to day 7. On days 7–10, mice of all the group were fed with water. Body weights of mice were measured every day, and disease activity index was evaluated by the the Waltham Feces Scoring system on day 3, 5, 7, and 9. Mice in each group were sacrificed on day 10, and the colon length of mice in each group of mice was measured. Colon tissues in each group were collected for testing inflammatory factors via an ELISA kit, and H&E straining was performed to observe histopathological changes of colon tissues.

### Sequencing Analysis

Colon samples of inflammatory bowel disease (IBD) mice in each group were collected after treatment. After 72 h of different delivery of *C. butyricum* in the groups of PBS, DSS, ETEC, N3, and N3-DBCO, mice were sacrificed, and colon samples were collected for 16S rDNA sequencing to quantify the amount of *C. butyricum* in the gut.

### Statistical Analysis

The experimental data were expressed as means ± SEM. The *in vitro* experiments were performed with at least three independent experiments. For *in vivo* experiments, animals were randomly divided into different groups with three to six mice. Statistical analysis of samples was conducted by one-way analysis of variance (ANOVA) or Student’s *t* test of GraphPad Prism 7.0.

## Supporting Information Available

Figure S1. *In vitro* bacterial biological properties after azido and DBCO modification. Figure S2. Zeta potential of bacteria after bacterial adhesion. Figure S3. *In vitro* bacterial adhesion between bioorthogonal group-modified *E. coli* MG1655 and *P. anaerobius*. Figure S4. TEM images of bioorthogonal group modified *E. coli* MG1655 and *P. anaerobius*. Figure S5. *In vitro* bacterial adhesion between bioorthogonal group modified *E. coli* MG1655 and *S. aureus*. Figure S6. *In vitro* characterization of bacterial aggregation at different bacterial densities. Figure S7. *In vivo* long-term colonization of bacteria in the GI tract of mice at different time points. Figure S8. *In vitro* colonization of bacteria in the GI tract of mice at different time points. Figure S9. DSS-induced colitis of mice with 3% DSS in drinking water for 7 consecutive days. Figure S10. *In vivo* bioorthogonal-mediated therapy in DSS-induced colitis mice model with *E. coli* Nissle1917. Figure S11. Bacterial counting by bacterial colony-forming units of ETEC in chromogenic *E. coli* agar plates (*n* = 3) (PDF)
