# Supplementary material for: In Situ Bioorthogonal Conjugation of Delivered Bacteria with Gut Inhabitants for Enhancing Probiotics Colonization
Source: ACS Cent Sci. 2022 Aug 25;8(9):1306–17. doi: 10.1021/acscentsci.2c00533 (PMC9523781; doi:10.1021/acscentsci.2c00533)
Supplement: Supplementary file 1 — oc2c00533_si_001.pdf [file oc2c00533_si_001.pdf]

Supporting information of

In situ bioorthogonal conjugation of delivered bacteria with gut inhabitants for enhancing probiotics colonization

Wen-Fang Song,[†,§] Wei-Qin Yao,[†,§] Qi-Wen Chen,[†] Diwei Zheng,[†] Zi-Yi Han,[†] Xian-Zheng Zhang[†,*]

[†]Key Laboratory of Biomedical Polymers of Ministry of Education & Department of Chemistry, Wuhan University, Wuhan 430072, P. R. China

* Corresponding author. E-mail: xz-zhang@whu.edu.cn

[§] Wen-Fang Song and Wei-Qin Yao contributed equally to this work.

This PDF file includes:

Figure S1 to S11

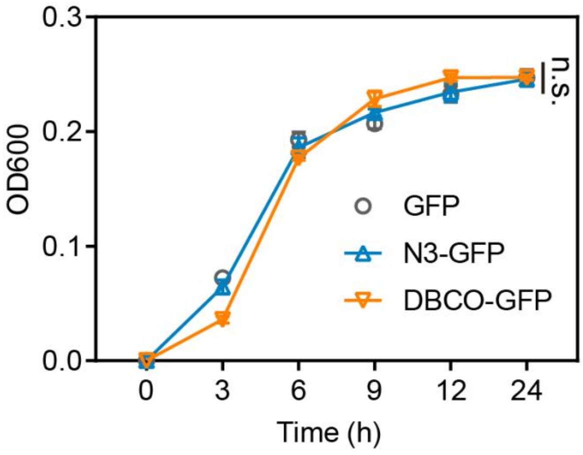

Figure S1. *In vitro* bacterial biological properties after azido and DBCO modification. The OD600 changes of GFP, azido-modified GFP and DBCO modified GFP during 24 hours. Significant differences were assessed in this figure using one-way ANOVA (Turkey; n. s., not significant)

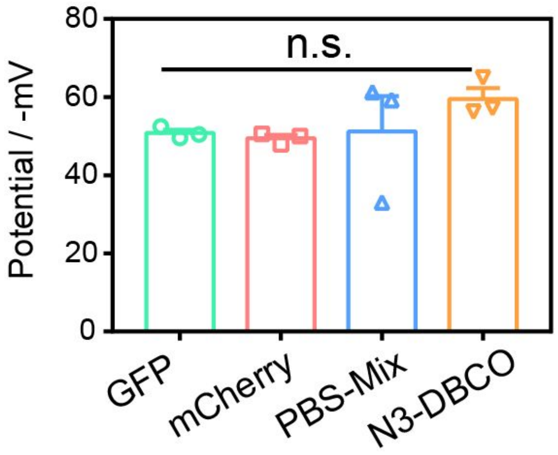

Figure S2. Zeta potential of bacteria after bacterial adhesion (n = 3). Significant differences were assessed in this figure using one-way ANOVA (Turkey; *n. s.*, not significant)

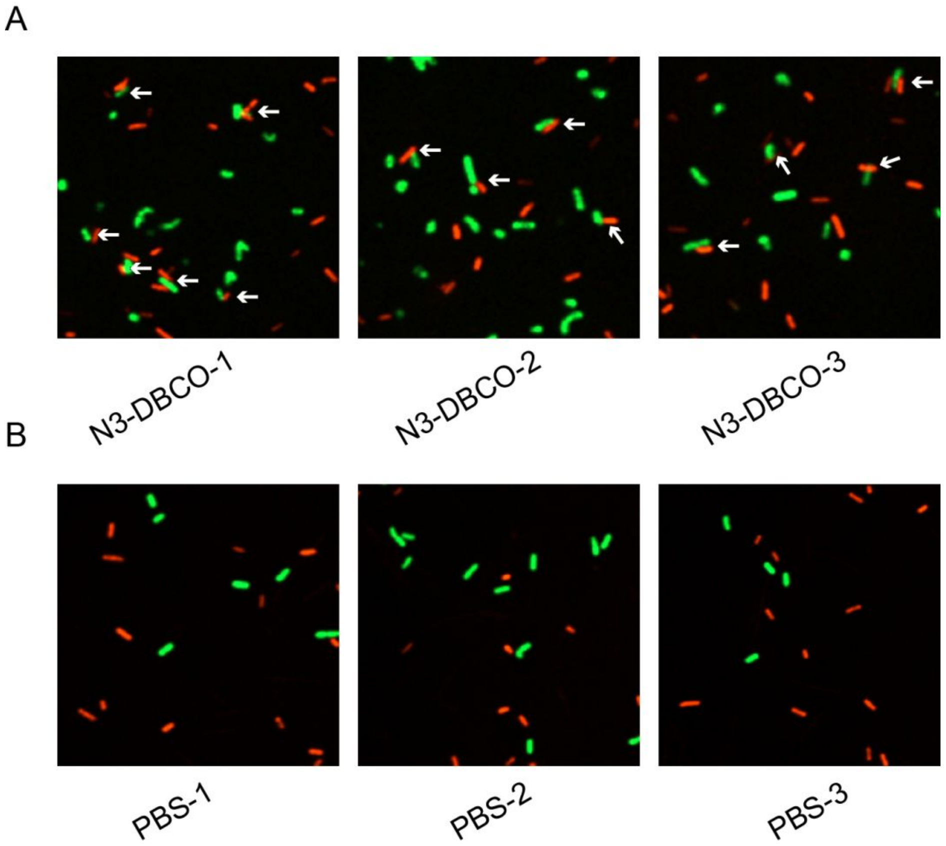

Figure S3. *In vitro* bacterial adhesion between bioorthogonal groups modified *E. coli* MG1655 and *P. anaerobius*. (A), Confocal fluorescence images of azide modified *E. coli* MG1655 (Cy5 labeled) after incubation with DBCO-decorated *P. anaerobius* (FITC labeled) for 2 h (n = 3). (B), Confocal fluorescence images of *E. coli* MG1655 (Cy5 labeled) after incubation with *P. anaerobius* (FITC labeled) for 2 h (n = 3).

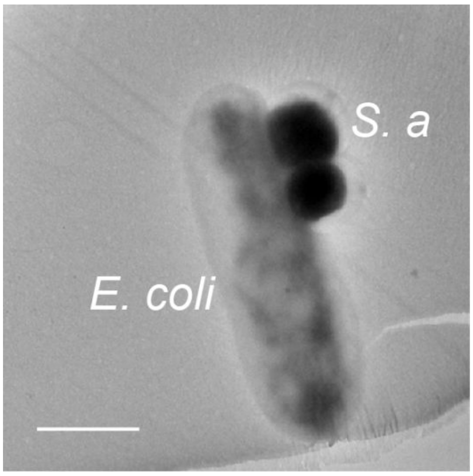

Figure S4. TEM images of bioorthogonal groups modified *E. coli* MG1655 and *P. anaerobius.* TEM image of bacterial adhesion of azide decorated MG1655 (N3-MG1655) and DBCO decorated *P. anaerobius* (DBCO-*P. a*). Scale bar, 1 μm

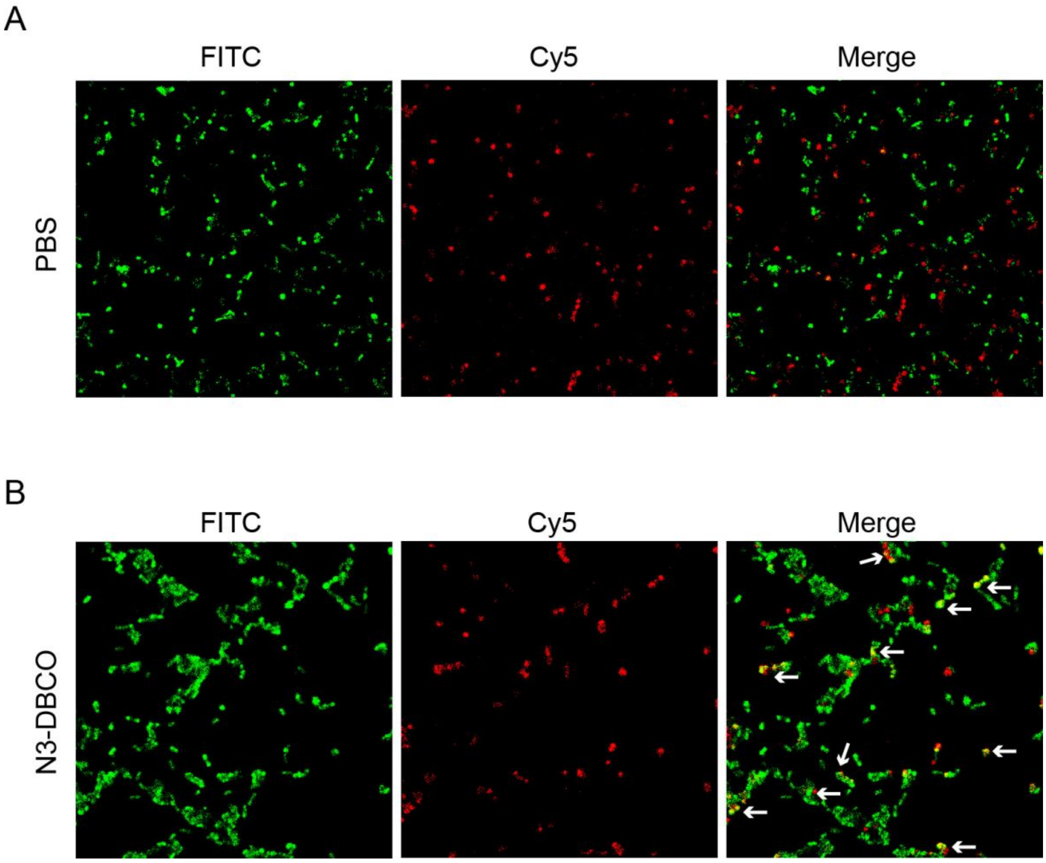

Figure S5. *In vitro* bacterial adhesion between bioorthogonal groups modified *E. coli* MG1655 and *S. aureus*. (A), Fluorescence colonization assay of azide-modified *S. aureus* (FITC labeled) and DBCO-functionalized *E. coli* MG1655 (Cy5 labeled). (B), Representative confocal fluorescence images of *E. coli* MG1655 (Cy5 labeled) after incubation with DBCO decorated *S. aureus* (FITC labeled) for 2 h (n = 3).

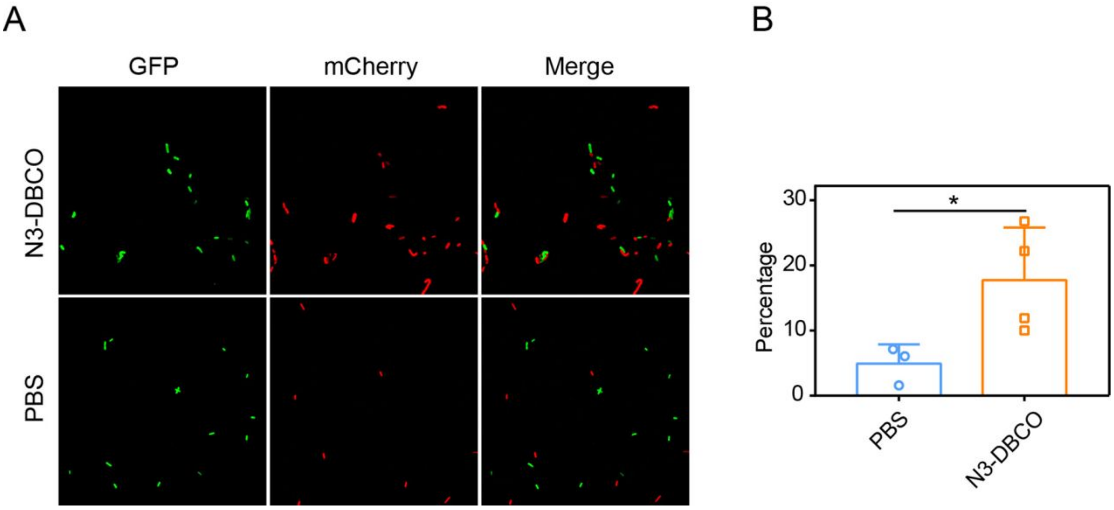

Figure S6. ***In vitro* characterization of bacterial aggregation at different bacterial densities.** (A), Representative confocal fluorescence images of 1:1 mixed cocultures at a bacterial density of OD = 0.06. (B), Aggregation ratio of bacterial aggregation in N3-DBCO and PBS groups (n = 3). Significant differences were assessed in (B) using t test (*P ≤ 0.05). The mean values and SEM are presented.

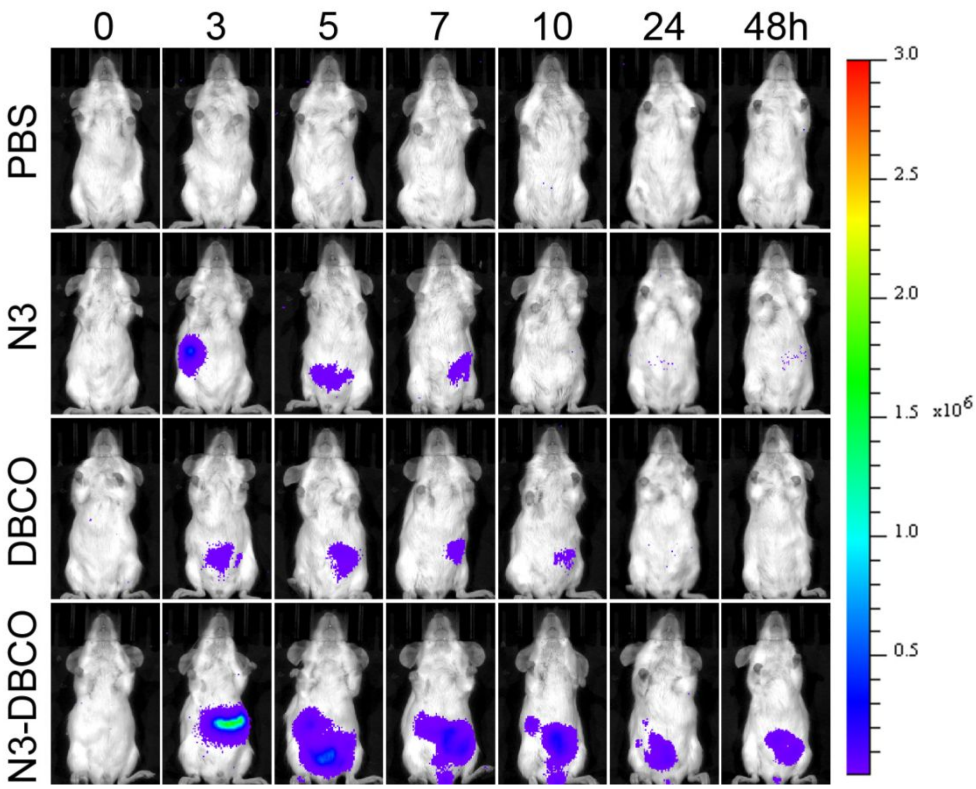

Figure S7. ***In vivo*** **long-term colonization of bacteria in GI tract of mice at different time points.** Representative IVIS images for evaluating bacterial retention in the GI tract of mice mediated by biorthogonal reactions (n = 3).

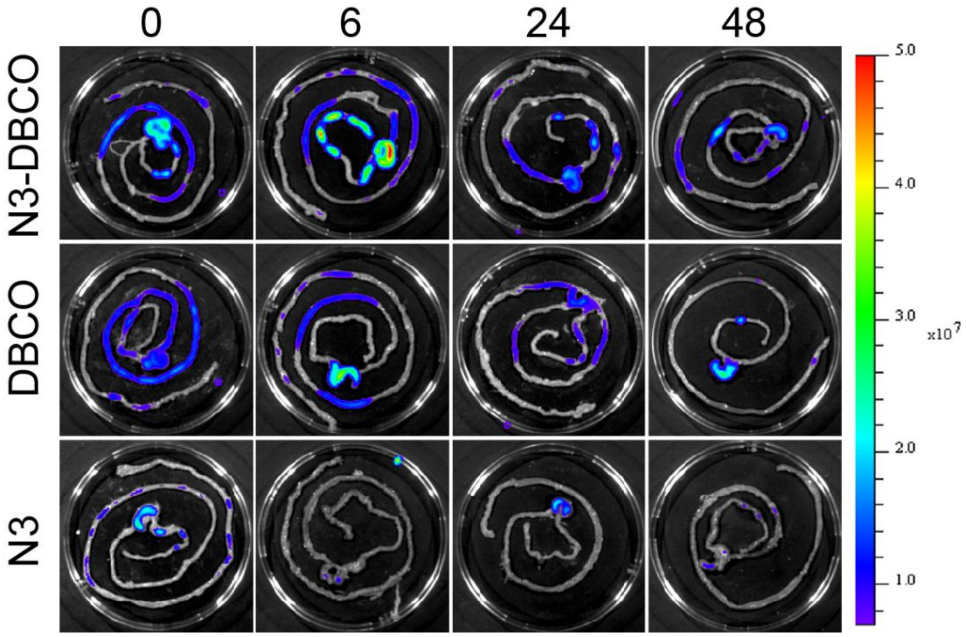

Figure S8. ***In vitro*** **colonization of bacteria in GI tract of mice at different time points.** Representative IVIS images of mice colons in each group for evaluating bacterial retention (n = 3).

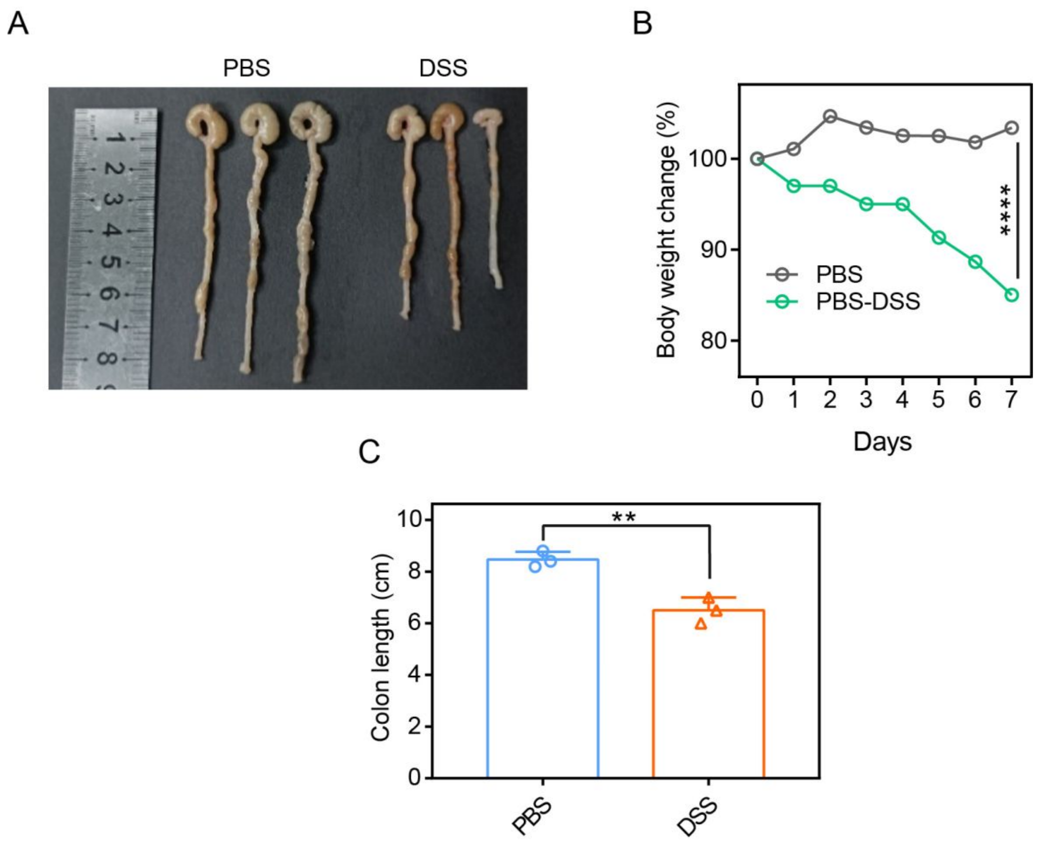

Figure S9. DSS induced colitis of mice with 3 % DSS in drinking water for 7 consecutive days. (A), Representative photographs of mice colon in DSS and PBS groups, respectively (n = 3). (B), Body weight changes over 7 days (n = 3). (C), Colon length of mice in each group (n = 3). Significant differences were assessed in (B) and (C) using t test (**P ≤ 0.01; ****P ≤ 0.0001). The mean values and SEM are presented.

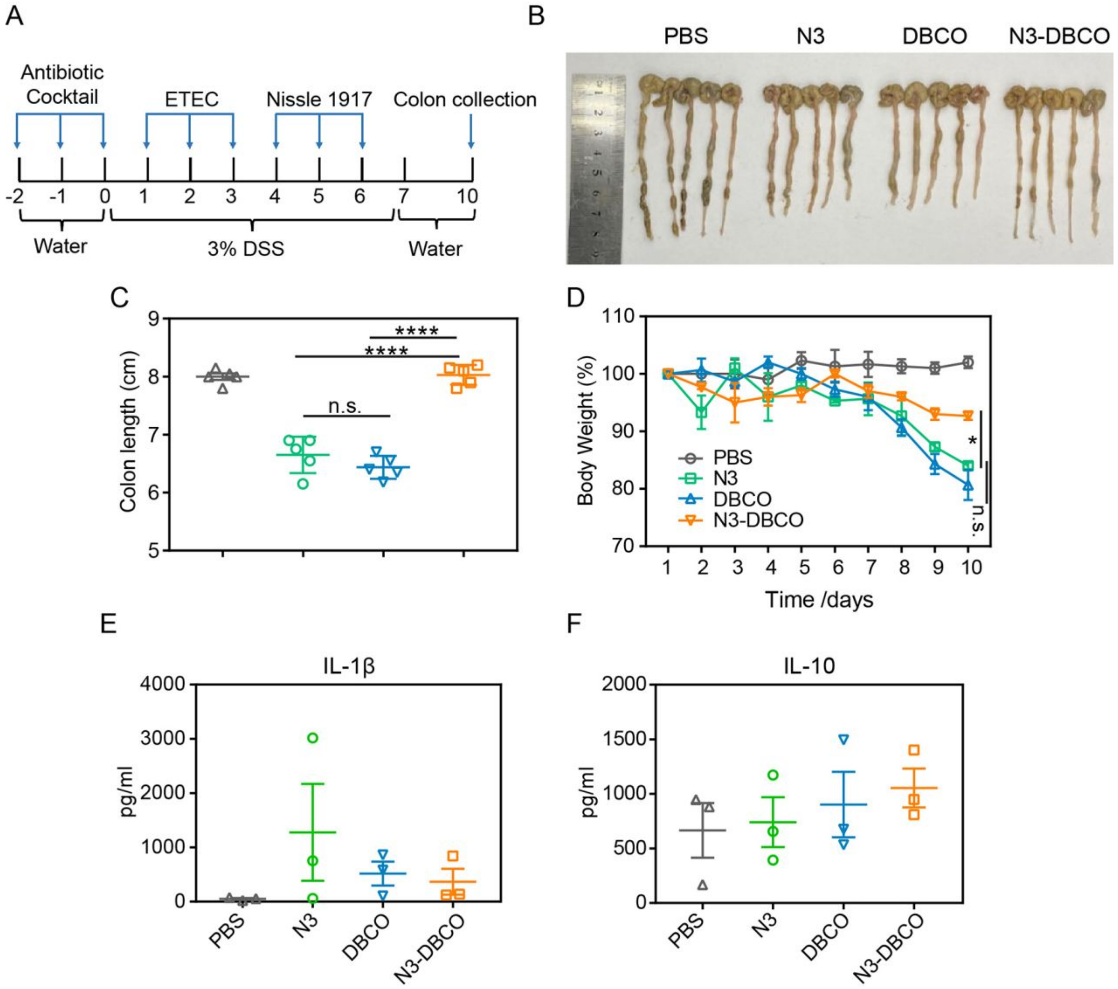

Figure S10. *In vivo* bioorthogonal-mediated therapy in DSS-induced colitis mice model with *E. coli* Nissle1917. (A), Schematic of administration schedule. C57BL/6 mice were provided with PBS, ETEC (10^8 CFU per mouse) or N3-ETEC (10^8 CFU per mouse) for 3 days after antibiotic cocktail treatment. On days 4, 5 and 6, mice were orally administered with PBS or *E. coli* Nissle1917 (10^8 CFU per mouse), DBCO modified *E. coli* Nissle1917 (10^8 CFU per mouse). Mice were euthanized on day 10. (B), Representative photographs of mice colon with different treatments on day 10 (n = 5). (C), Colon length of mice in each group (n = 5). (D), Body weight changes (n = 5). (E) and (F), IL-10 and IL-1β proteins in colon tissue homogenates by ELISA (n = 3). Significant differences were assessed in (C) using one-way ANOVA and using t test in (D) (*$P \leq 0.05$; ****$P \leq 0.0001$; n.s., not significant). The mean values and SEM are presented.

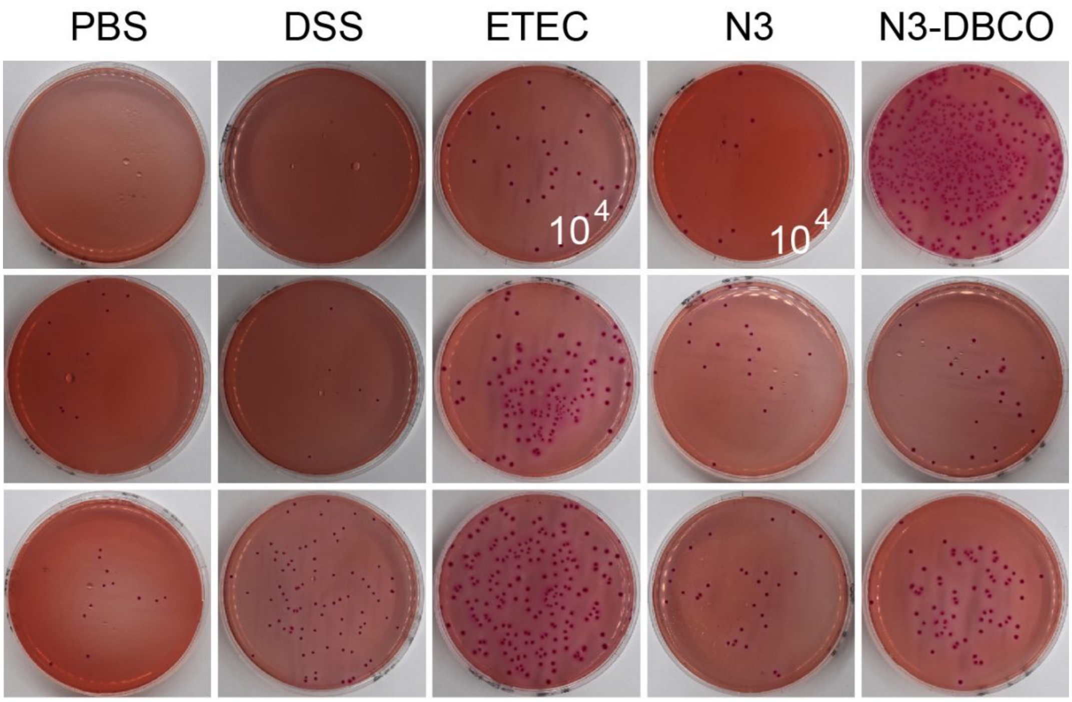

Figure S11. Bacterial counting by bacterial colony-forming units of ETEC in chromogenic *E. coli* agar plate (n = 3).
